# Zolpidem for the Treatment of Dystonia

**DOI:** 10.3389/fneur.2019.00779

**Published:** 2019-07-17

**Authors:** Stephanie Patricia J. Badillo, Roland Dominic G. Jamora

**Affiliations:** ^1^Department of Clinical Neurosciences, University of the East Ramon Magsaysay Memorial Medical Center, Quezon City, Philippines; ^2^Section of Neurology, Department of Internal Medicine, Cardinal Santos Medical Center, San Juan City, Philippines; ^3^Movement Disorder Service and Section of Neurology, Institute for Neurosciences, St. Luke's Medical Center, Quezon City, Philippines; ^4^Department of Neurosciences, College of Medicine – Philippine General Hospital, University of the Philippines Manila, Manila, Philippines

**Keywords:** zolpidem, treatment, dystonia, movement disorders, systematic review

## Abstract

**Background and Purpose:** There are recent reports of zolpidem being effective for the treatment of a variety of movement disorders, due to its action on the gamma-aminobutyric acid A receptors in the thalamus, subthalamic nucleus, and globus pallidus, hence facilitating inhibitory pathways in the basal ganglia motor loop. Its beneficial effects have been described for Parkinson's disease and other related disorders. The objective of this study was to assess the therapeutic effects of zolpidem for various types of dystonia.

**Methods:** We conducted a literature search using MEDLINE via PubMed, Cochrane Library, EMBASE, Scopus, and Google Scholar.

**Results:** There were no randomized controlled trials. The literature included 6 case reports, 4 case series, and 1 non-randomized, non-controlled interventional trial. Overall, 49 adult participants (range 1–34 participants) with a mean age of 49.5 years were treated. Regardless of the dystonia subtype, a single dose of zolpidem at 10 mg causes improvement of symptoms for a mean duration of 3.4 h until patient returns to baseline. The main adverse effect noted was drowsiness, which was dose-dependent.

**Conclusion:** While the current available literature suggests that zolpidem may be an effective pharmacologic option for treating dystonia, however the quality of evidence remains limited. Larger sample size, methodological consistency, and randomized controlled trials with long-term patient follow-ups are necessary to come up with definitive conclusion.

## Introduction

Dystonia is a hyperkinetic movement disorder characterized by sustained or intermittent muscle contractions causing abnormal, often repetitive, movements, postures, or both. Movements are typically patterned, twisting, and may be tremulous. It is often initiated or worsened by voluntary action and associated with overflow muscle activation (1). Estimation of overall prevalence of dystonia is difficult due to variations in age and ethnicities of study population as well as underreporting of cases (2, 3). Dystonia may be chronic and progressive, especially primary dystonias which are associated with genetic mutations and often begin in younger age groups (1, 3). Thus, dystonia can cause significant disability and impairment in quality of life.

There are several treatment options for dystonia, including pharmacological therapies such as the use of anticholinergics (most commonly trihexyphenydil), botulinum toxin injections, and surgical treatment such as deep brain stimulation (DBS) of the globus pallidus interna (4–6). However, the medical treatment of dystonia remains difficult and often unsuccessful. This is often frustrating for patients who suffer from chronic and refractory forms of dystonia, such as those with X-linked dystonia parkinsonism (XDP) (7).

Zolpidem is an imidazopyridine with a chemical formula of *N,N,6-trimethyl-2-(4-methylphenyl)-imidazo[1,2-*α]-*pyridine-3-acetamide hemitartrate* (8). It binds to the α_1_ subunits of gamma-aminobutyric acid (GABA)_A_ receptors in the central nervous system, facilitating inhibitory neurotransmission (8, 9). It is a sedative-hypnotic for patients with sleep disorders, and also has minor anxiolytic, muscle relaxant, and anticonvulsant properties (8). Several recent reports have demonstrated the beneficial effect of zolpidem in treating various types of dystonia. The objective of this review was to describe the therapeutic effects of zolpidem on dystonia based on the available published literature. This review does not limit based on subtype of dystonia.

## Materials and Methods

### Eligibility Criteria

We included studies that reported or investigated the use of zolpidem for any type of dystonia, published on or before September 30, 2018. Studies were excluded if they were not in English, were animal studies or were not from the primary literature (such as reviews and commentaries). Since very few of these studies have been done, no limits were placed on study design, patient demographic variables, or outcome measures.

### Search Strategy

Searches were run in MEDLINE via PubMed, the Cochrane Library, EMBASE, Scopus, and Google Scholar. Currently, there are no zolpidem trials registered with clinicaltrials.gov. All searches were run on or before September 30, 2018.

### Study Selection

The studies were screened by the primary investigator (SPB). Any study that used zolpidem to treat dystonia was included in the next stage of the review. A second round of screening was performed by the authors based on abstract and/or full text and studies that met the eligibility criteria above were included. Reference lists of all studies identified during the first round of screening were cross-checked for additional studies that would meet the eligibility criteria. The Preferred Reporting Items for Systematic Reviews and Meta-analysis (PRISMA) statement guidelines were followed (10).

### Data Collection

Data items included study design, diagnosis, country of first author's institution, sample size, total number of participants, mean age and sex of study participants, mean duration of diagnosis, outcome measures, dose of zolpidem, number improved, duration of effect, and adverse effects. No assessment for risk of bias was performed since the accepted tools for this type of assessment are designed primarily for randomized controlled trials (RCTs). This review includes all study designs and hence these tools would not be appropriate. Instead, data was collected on randomization, controls and masking (if available), as well as informal notes on each study's limitations.

### Outcome Measures

Majority of the studies (*n* = 8/11, 73%) described outcomes using clinical observation only (11–18). Three studies used the Burke-Fahn-Marsden Dystonia Rating Scale (BFMDRS) as a measure of outcome (5, 6, 19). The BFMDRS is a rating scale that reliably indicates the severity of primary dystonias (20, 21).

Out of the 3 studies included in this review that used the BFMDRS as an outcome measure, only 1 study defined degrees of improvement (5). This was an interventional trial in which more than 40% improvement in BFMDRS was defined as “remarkable improvement,” <40% improvement as “mild improvement,” and no change in the scale as “no improvement” (5).

## Results

**Figure 1:**
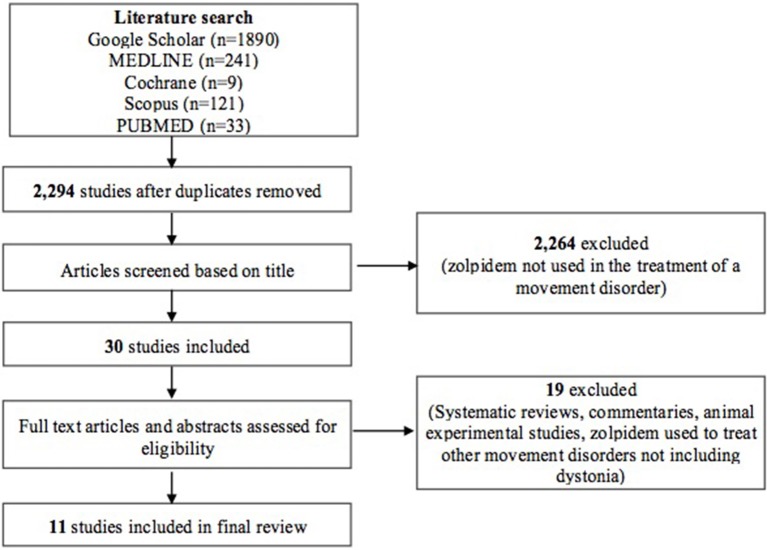
PRISMA flow diagram illustrating selection of studies.

Our initial search using a combination of the keywords “zolpidem” AND (“dystonia” OR “dyskinesia” OR “movement disorders”) produced 2,294 studies after duplicates were removed. Individual database results included Google Scholar (*n* = 1,890), MEDLINE (*n* = 241), Cochrane (*n* = 9), Scopus (*n* = 121), and PUBMED (*n* = 33). Thirty studies were included after initial screening based on the title only. A second round of screening was done by applying eligibility criteria to study abstracts and/or full texts. Systematic reviews, commentaries, experimental studies using animal subjects and other studies that used zolpidem for the treatment of other movement disorders not including dystonia [such as Parkinson disease (PD), Progressive Supranuclear Palsy (PSP)] were excluded from this review (see Figure 1). A total of 11 studies were finally included for full review.

### Study Designs

There were 7 case reports (12–18) and 4 case series (having at least 2 participants) (5, 6, 11, 19). There was only 1 interventional trial, and this was also the only study that had more than 10 participants (5). This was a non-randomized, non-controlled study among 34 patients with dystonia who were refractory to medications (such as clonazepam, baclofen, and trihexyphenydyl). Once placed on zolpidem, the doses of their other medications were unchanged.

### Participant Characteristics

The total number of adult participants was 49, with 30 males (61.2%). One study did not mention the patient's gender (13). The number of participants per study ranged from 1 to 34, with most of the studies (*n* = 7, 64%) having only one participant. The ethnicity of the subjects was often not reported; however, studies were conducted in 5 countries: Korea (*n* = 4), USA (*n* = 4), Japan (*n* = 2), and Argentina (*n* = 1). Table 1 includes the mean age and sex of participants, subtype of dystonia, the duration of illness, as well as the countries where each study was conducted.

**Table 1:** Characteristics of studies included in the review.

**Author**	**Country**	**Diagnosis**	**Age (years)**	**Sex**	**Time since diagnosis (years)**	**Dose per administration (mg)**	**Average total daily dose (mg)**	**Outcome**	**Improved/ Total**	**Duration of effect (h)**	**Adverse effects**
Evidente (19)	USA	X-linked dystonia parkinsonism (“lubag”)	41	M	2	10	80–100	BFMDRS, UPDRS	3/3	2	Sedation if more than 20 mg daily
			36	M	1	10	20			3	
			38	M	1	10	20			2	
Garreto et al. (11)	Argentina	Meige syndrome	57	F	11	10	70	Observation	3/3	3	None
		Blepharospasm	63	M	8	10	30			3	Mild sedation
		Meige syndrome	66	M	22	10	20			3	None
Vazquez-Delgado and Okeson (12)	USA	Oromandibular dystonia	59	F	5	10	10	Observation	1/1	N/A	None
Seo and Jeong (13)	Korea	Dystonia - segmental axial	35	N/A	2	10	10	Observation	1/1	N/A	Mild visual dimness
An et al. (14)	Korea	Meige syndrome	59	M	11	12.5 (CR)	50	Observation	1/1	4	None
Park et al. (15)	Korea	Myoclonus-dystonia syndrome	36	F	16	10	40	Observation	1/1	6	None
Sunwoo (16)	Korea	Blepharospasm	74	F	20	10	10	Observation	1/1	5	None
Miyazaki et al. (5)	Japan	Generalized dystonia	38.3 ± 19.4	3M/6F	4.6 ± 6.8	10	12.2 ± 6.	BFMDRS	3/9	N/A	Sedation (8 out of 34 total patients, 3 out of 8 zolpidem responders)
		Meige syndrome/ blepharospasm	60.6 ± 9.6	6M/4F	3.6 ± 3.2	10	12.0 ± 4.8		2/10		
		Cervical dystonia	45.7 ± 14.4	7M/0F	6.0 ± 4.9	10	10 ± 0		0/7		
		Hand dystonia	48.4 ± 10.1	5M/3F	7.4 ± 5.2	10	8.8 ± 5.1		3/8		
Waln and Jankovic (17)	USA	Blepharospasm	67	F	17	10	25	Observation	1/1	N/A	None
Miyazaki et al. (6)	Japan	Isolated dystonia	35	M	15	10	20	BFMDRS	2/2	4	Sedation if 20 mg single dose was used
			20	M	6	10	20			3	
Martinez-Ramirez et al. (18)	USA	Oromandibular dystonia	52	M	3	10	15	Observation	1/1	3	None

*CR, zolpidem continuous release formulation; N/A, information not available or not reported; BFMDRS, Burke-Fahn-Marsden Dystonia Rating Scale; UPDRS, Unified Parkinson's Disease Rating Scale*.

### Treatment Outcomes

Out of a total of 49 adult patients included in this review, 25 patients (51%) showed almost complete resolution of symptoms immediately after intake of zolpidem 10 mg, with an onset of effects being observed in 15–45 min and peak effects in 1–2 h (5, 6, 11–19). The dose per administration ranged from 5 to 10 mg, with most patients being given the 10 mg dose. The mean duration of effect of zolpidem was 3.4 ± 2.5 h. The longest duration of action observed for a single 10 mg dose of zolpidem was 6 h, given to a 36-year-old female with myoclonus-dystonia syndrome (15).

Because of the short duration of action of zolpidem, dosing varied from once daily to four times daily, with a mean total daily dose of 30 mg, usually given at 10 mg three times daily. Doses of 20 mg daily or higher were divided into three to four times per day to avoid the side effect of somnolence (14, 15). The highest total daily dose tolerated was 80–100 mg (given at 10 mg every 2 h), which was administered to a 41-year-old male with chronic XDP who was a long-term user of the medication (19). One study used the controlled release (CR) formulation in order to achieve a slightly more prolonged effect of 4 h (14). No dose modifications were done based on age or sex. There was no mention of any hepatic or renal impairment that would necessitate dose adjustment of zolpidem.

In the three studies that used the BFMDRS scores as an outcome measure, a significant improvement was seen after zolpidem therapy in various types of dystonias (5, 6, 19). After therapy with zolpidem at 5–20 mg, the BFMDRS scores in patients suffering from various types of dystonia were significantly decreased from 7.2 ± 7.9 to 5.5 ± 5.0 (5). There was improvement among patients with generalized dystonia, Meige syndrome/blepharospasm and hand dystonia (by 27.8, 17.8, and 31.0%, respectively). Notably, there was no improvement seen among 10 patients with cervical dystonia (5). A patient with isolated dystonia with a baseline BFMDRS score of 57 improved in a dose-dependent manner, to a score of 27 after intake of zolpidem 10 mg, and to a score of 19 after intake of zolpidem 20 mg (6). Improvement was noted within 20–30 min of drug administration, was optimal after 2 h, and lasted for 4 h (6). In a case series of three Filipino male patients with XDP, the patients (mean age of 38 years) showed a mean of 54% improvement of dystonia and a mean of 25% improvement in parkinsonism, at a dose of zolpidem 10 mg (19).

There was also noted improvement in dystonias unresponsive to other medications. A case report of a patient with Meige syndrome, initially unresponsive to botulinum toxin A, baclofen, and trihexyphenidyl, showed complete resolution of symptoms within 1 h of intake of zolpidem 12.5 mg, with symptom free periods lasting for 4 h (14). A patient with blepharospasm refractory to botulinum toxin injections showed much improvement of symptoms within 30 min after taking zolpidem 10 mg, with effects lasting for 5 h (16).

## Discussion

Zolpidem is a sedative-hypnotic initially approved for the treatment of sleep disorders. It was first marketed in Europe in 1987. In April 1992, it was approved by the United States Food and Drug Administration as a hypnotic for patients with sleep disorders, with a recommended dose of 5–10 mg to be taken at bedtime (8, 22). Since then, there have been case reports of patients with different neurologic disorders responding to zolpidem therapy. In 1997, a case report noted that a woman with PD experienced significant improvement in her rigidity and akinesia after receiving zolpidem (23). In 2000, there was a case report of a South African man who was in a persistent vegetative state for 3 years after a motor vehicle crash, who suddenly awoke and began communicating with his family 15 min after receiving zolpidem (24). In both cases, the patients returned to baseline after a few hours, however the effects were reproducible with repeated use of the drug. At present, there are numerous case reports of patients with various neurologic disorders such as PD (25–30), PSP (31–36), or those in minimally conscious states (24, 37) whose symptoms have improved in various degrees with the use of zolpidem.

In particular, our findings showed that among patients with dystonia, a single dose of zolpidem rapidly improved symptoms within 15–45 min, peaking at 1–2 h. However, effects were short-lived lasting up to 3–4 h. These findings are reproducible among patients with various types of primary dystonia, regardless of age or sex. The duration of effect is similar to reports describing the effects of zolpidem among patients with PD (23, 25–30) and PSP (31–36). These are also consistent with the published literature on the pharmacokinetics of zolpidem among healthy adult subjects. It has a rapid mean time to reach peak plasma concentration (t_max_) of 0.75–2.6 h postdose, an oral absolute bioavailability of 70%, and a short mean half life of 1.5–3.2 h (8).

Zolpidem is thought to improve the symptoms of patients with movement disorders through its facilitation of inhibitory pathways in the basal ganglia loop. Its binding site is located in the α subunit of the GABA_A_ receptor (9). The GABA_A_ receptor is a chloride ion channel with a pentameric structure, assembled from five subunits selected from multiple polypeptide classes (for example, α, β, γ, δ, etc.) (9). GABA_A_ receptors are classified according to their α subunit, which may have specific isoforms (α1, α2, α3, α4, α5, α6). The binding sites for zolpidem and for benzodiazepines is in the α subunit. Benzodiazepines can bind to multiple types of GABA_A_ receptors that contain α_1_, α_2_, α_3_, or α5. However, zolpidem binds selectively to GABA_A_ receptor isoforms that contain α1 subunits (4). Once the GABA_A_ receptor is then activated, the release of GABA facilitates inhibitory neurotransmission throughout the central nervous system (8).

In recent experimental studies, a high density of GABA_A_ receptors was found in the basal ganglia and thalamus (38, 39). In a single blind, placebo controlled study done in healthy volunteers with no history of drug abuse, acute oral administration of a therapeutic dose of zolpidem (10 mg) reduced thalamic GABA levels (38). Experimental studies among rat subjects showed that zolpidem enhances GABA transmission in the subthalamic nucleus (40) and in the globus pallidus (41). Hence, binding of zolpidem to the GABA_A_ receptor facilitates the effects of GABA throughout the basal ganglia motor loop, perhaps accounting for clinical improvement of dystonia (8).

There is limited data in the literature regarding the long term efficacy of zolpidem for the treatment of dystonia since reports do not usually mention follow ups. In a patient with isolated dystonia, the effect of zolpidem in reducing the symptoms of dystonia was sustained 6 months after the initial dose, with a reduction of the BFMDRS score to one-third of baseline (6). Among 3 patients with XDP, the efficacy of zolpidem was maintained in 2 patients after 6 months to 1 year, with one patient discontinuing the medication due to financial constraints (19).

More information is needed in order to determine optimum dosing. At present, zolpidem is currently available as an immediate release tablet (5 mg and 10 mg), an extended release tablet (6.25 mg and 12.5 mg), an oral spray (5 mg/spray), and a sublingual tablet (1.75, 3.5, 5, and 10 mg) (22). Most studies in this review used a total of 30 mg in divided doses due to short duration of action and to avoid somnolence. As such, the lowest effective daily dose is recommended. In the available literature, lower doses of zolpidem are recommended for elderly patients and those with renal and hepatic impairment (8). In elderly men, oral clearance of zolpidem is one third compared to younger men and the half life is doubled. A similar difference, though less pronounced was seen among elderly women as compared to younger women (42). In patients with renal disease, there is an increased fraction of unbound zolpidem in plasma, while patients who undergo regular hemodialysis have a slower rate of elimination of zolpidem as compared to healthy adults (42). Among those with liver cirrhosis, there is also an increased fraction of unbound zolpidem in plasma, with a 3-fold increase in half life as well due to impaired hepatic clearance.

The main adverse effect of zolpidem as described in the literature was drowsiness (when it is not being prescribed as a sedative). Daytime somnolence was observed among patients taking a single dose of zolpidem 20 mg or more in the morning (5, 6, 19). Drowsiness was not observed among patients taking single doses of 5–10 mg, those who had higher daily doses of 20–40 mg but in divided doses (9, 13) or those taking the CR formulation (14). This finding was similar to that of case reports describing the effects of zolpidem as treatment for patients with PD (26, 28). Other adverse effects described in the literature were amnesia and abnormal behavior (somnambulism) (43). Falls, confusion and memory disorders were associated with the following risk factors: increased dose of the drug (10 mg or more), inpatients, elderly patients (aged 80 years and older), presence of gait and balance disorders, dementia, and concomitant drugs (43). Hence, an initial 5 mg dose was recommended in the elderly. This is consistent with results of earlier studies on the pharmacokinetic properties of zolpidem, which showed that elderly men and women over the age of 70 had markedly lower clearances and volumes of distribution (8, 42).

There were no studies that described the adverse effects of long term use of zolpidem among patients with dystonia. However, these have been extensively described in studies among patients with sleep disorders. Zolpidem is not known to cause hepatic, respiratory, cardiovascular, or renal dysfunction (43). No effects on cognitive function nor complications during pregnancy and childbirth have been reported (43).

## Conclusion

While the available literature suggests that zolpidem may be an effective pharmacologic option for treating dystonia, the quality of evidence remains limited since the studies available were case reports and small observational studies only. Regardless, the potential of zolpidem to improve quality of life and functional outcome especially among patients with medically refractory and chronic dystonias merits further investigation with additional, larger-scale, standardized controlled trials.

## Data Availability

All datasets generated for this study are included in the manuscript.

## Author Contributions

SB: study concept and design, acquisition of data, analysis and interpretation and writing of the initial draft. RJ: study concept and design, acquisition of data, analysis and interpretation, critical revision of the manuscript for intellectual content, and study supervision.

### Conflict of Interest Statement

RJ serves on the advisory board of Lundbeck Phils and Torrent Phils. He has received honoraria and CME grants from the Philippine offices of Allergan, Innogen, Lundbeck, Medichem, Natrapharm, Sandoz, Sun, Torrent, and Zydus. He has research grants from the Collaborative Center for X-linked Dystonia Parkinsonism and the Philippine Neurological Association. The remaining author declares that the research was conducted in the absence of any commercial or financial relationships that could be construed as a potential conflict of interest.
